# Development of a Pediatric Ebola Predictive Score, Sierra Leone^1^

**DOI:** 10.3201/eid2402.171018

**Published:** 2018-02

**Authors:** Felicity Fitzgerald, Kevin Wing, Asad Naveed, Musa Gbessay, J.C.G. Ross, Francesco Checchi, Daniel Youkee, Mohamed Boie Jalloh, David E. Baion, Ayeshatu Mustapha, Hawanatu Jah, Sandra Lako, Shefali Oza, Sabah Boufkhed, Reynold Feury, Julia Bielicki, Elizabeth Williamson, Diana M. Gibb, Nigel Klein, Foday Sahr, Shunmay Yeung

**Affiliations:** University College London Great Ormond Street Institute of Child Health, London, UK (F. Fitzgerald, N. Klein);; Save the Children, Freetown, Sierra Leone, and London (F. Fitzgerald, K. Wing, A. Naveed, M. Gbessay, J.C.G. Ross, F. Checchi);; London School of Hygiene & Tropical Medicine, London (K. Wing, F. Checchi, S. Oza, S. Boufkhed, E. Williamson, S. Yeung);; Kings Sierra Leone Partnership, Kings Centre for Global Health, Kings College London, London (D. Youkee);; 34 Military Hospital, Republic of Sierra Leone Armed Forces, Freetown (M.B. Jalloh, F. Sahr);; Ola During Children’s Hospital, Sierra Leone Ministry of Health, Freetown (D.E. Baion, A. Mustapha);; Cap Anamur (German Emergency Doctors), Ola During Children’s Hospital, Freetown (H. Jah);; Welbodi Partnership, Ola During Children’s Hospital, Freetown (S. Lako);; Western Area Emergency Response Centre, Freetown (R. Feury);; MRC Clinical Trials Unit at UCL, London (J. Bielicki, D.M. Gibb);; Farr Institute of Health Informatics, London (E. Williamson)

**Keywords:** Ebola virus, Ebola virus disease, viral hemorrhagic fever, children, pediatrics, case definition, child mortality, prediction, viruses, Sierra Leone

## Abstract

We compared children who were positive for Ebola virus disease (EVD) with those who were negative to derive a pediatric EVD predictor (PEP) score. We collected data on all children <13 years of age admitted to 11 Ebola holding units in Sierra Leone during August 2014–March 2015 and performed multivariable logistic regression. Among 1,054 children, 309 (29%) were EVD positive and 697 (66%) EVD negative, with 48 (5%) missing. Contact history, conjunctivitis, and age were the strongest positive predictors for EVD. The PEP score had an area under receiver operating characteristics curve of 0.80. A PEP score of 7/10 was 92% specific and 44% sensitive; 3/10 was 30% specific, 94% sensitive. The PEP score could correctly classify 79%–90% of children and could be used to facilitate triage into risk categories, depending on the sensitivity or specificity required.

The Ebola virus disease (EVD) outbreak in West Africa claimed >11,000 lives with nearly 30,000 cases (*1*). During the outbreak in Sierra Leone, patients arriving at healthcare facilities were screened for EVD using World Health Organization (WHO) case definitions. Those fulfilling the case definition for suspected EVD were admitted to Ebola holding units (EHUs) to have blood taken for EVD testing and receive medical care until test results were available (Technical Appendix Figure 1). Testing was usually performed offsite, with a turnaround time for results of ≈48 hours (*2*). During admission, however, EVD-negative patients risked exposure to EVD, raising concerns that EHUs could act as amplification sites for infection (*3*–*7*). Children, many of whom were unaccompanied, were particularly vulnerable, and, because EHUs were overstretched, supervision to minimize the risk of cross-infection was challenging (*4*,*8*).

An accurate case definition for suspected EVD is critical for future outbreaks. Insufficient sensitivity of case definitions results in EVD-positive patients not being isolated, risking onward transmission in the community. There is an inherent tension between the public health priority to maximize the sensitivity of the case definition (minimizing onward transmission risk) and the individual patient’s perspective. The trade-off made by lower specificity means that many EVD-negative patients are kept waiting in EHUs for test results, risking nosocomial infection and delaying treatment for their true underlying condition. Case definitions should be flexible because priorities may change as outbreaks progress. In the 2014–2015 epidemic, the proportion of patients testing positive decreased over time: in October 2014, 77% of those admitted to a Freetown EHU tested positive, versus 1% in April 2015 (*5*).

In Sierra Leone, 2 case definitions were used for suspected EVD (*9*). Until November 2014, most EHUs used a WHO case definition that was the same for both adults and children, defining anyone who had >3 symptoms consistent with EVD and fever, or who had fever and had contact with a person with EVD, as having a suspected case (early-2014 case definition). Beginning in December 2014, the WHO case definition was modified to be age dependent (late-2014 case definition) (Figure 1; Technical Appendix Table 1). Under this definition, children only required fever and either 1 symptom (in children <5 years of age), 2 symptoms (in children 5–12 years of age), or >3 symptoms (in children >12 years of age) (*4*). This definition increased the likelihood of admitting EVD-negative children. Furthermore, in overstretched EHUs, children may have been admitted without meeting the criteria for suspected EVD, regardless of definition. In a mixed-age West African cohort, 9% of those admitted did not fulfill the early-2014 case definition (*3*).

**Figure 1:**
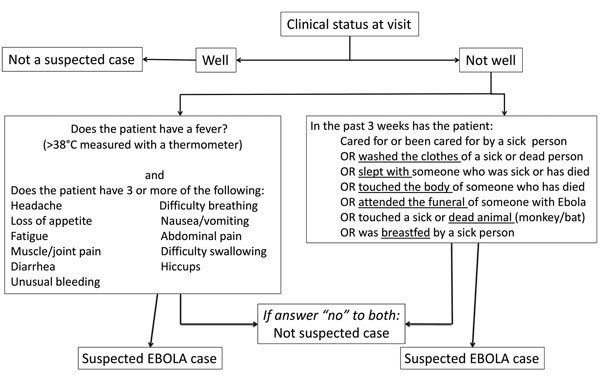
World Health Organization screening flowchart for Ebola virus disease used during outbreak in Sierra Leone (late-2014 case definition). Adapted from (*9*).

We aimed to develop a predictive score that could be used to tailor the pediatric case definition for suspected EVD according to the clinical and epidemiologic setting. The goal was to potentially limit unnecessary admissions to EHUs for EVD-negative children without reducing sensitivity.

## Methods

### Data Sources

We collected data on all children <13 years of age admitted to 11 EHUs in Sierra Leone (August 2014–March 2015) and built training and validation datasets. We performed multivariable logistic regression on the training dataset to generate a pediatric Ebola predictive (PEP) score, which we tested on the validation dataset. The age cutoff matched the WHO case definition distinguishing between children and adolescents, anticipating that adolescents would have an adult disease phenotype. Settings and data collection methods have been described previously (*4*,*10*). We visited each EHU to extract data from paper clinical records, case investigation forms, and site admission books and to interview staff. We cross-referenced data with the Western Area Ebola Response Centre (WAERC) database and 4 further sources, and single-entered data into a password-protected database (Epi Info version 7.1.4; US Centers for Disease Control and Prevention, Atlanta, GA, USA) (Technical Appendix). We removed personal identifiers before analysis and developed a schema for record matching across databases (Technical Appendix). We obtained ethics approval for this study from the Sierra Leone Ethics and Scientific Review Committee and the London School of Hygiene and Tropical Medicine Ethics committee (reference 8924).

### Statistical Analysis

We used Stata version 14.0 (StataCorp LLC, College Station, TX, USA) to perform analyses and limited analysis to children with EVD laboratory test result data. Variables were sex, age, contact history (yes/no), presence of 16 symptoms at EHU admission (yes/no), and days from symptom onset to EHU visit (*4*). We included age as a binary variable (<2 years and >2 years), given the higher burden of febrile illnesses that appear similar to EVD (e.g., malaria) in younger children. We considered data to be missing from the analysis if no value had been entered in the source documents (i.e., neither yes nor no).

Descriptive analysis of the cohort comprised the number of children with data available for each variable and the prevalence of signs and symptoms by laboratory-confirmed EVD status. We estimated the proportion of children (for whom we had sufficient data) who met the late-2014 WHO case definition.

### Predictive Model Building and Validation and Development of Risk Score

We split the data randomly into 2 datasets with equivalent proportions of laboratory-confirmed EVD-positive children: a training dataset for predictive score building, and a validation dataset to assess score performance (*11*). Using the training dataset, we calculated crude odds ratios (ORs) of association between potential predictive variables and outcome (laboratory-confirmed EVD status) and created an initial multivariable model including all potential predictive variables. A final training model was obtained by removing variables with p>0.3 from the fully adjusted model in a backward-stepwise fashion. The variables retained for constructing candidate PEP scores were age, gender, contact history, days from first symptoms to admission, and whether all symptoms were systematically documented (Technical Appendix).

We created the PEP score by assigning integer scores to variables in the validation dataset on the basis of their regression coefficients in the training dataset model (score = 1 for coefficients <1, score = 2 for coefficients >1) (*12*). We calculated each child’s overall PEP score by adding together the integer scores for the variables present, which resulted in possible PEP scores of 0–10. To identify the most clinically useful PEP score, we computed the sensitivity, specificity, positive predictive value, negative predictive value, and percentage of children correctly classified (compared with the standard of laboratory confirmation of EVD) of each candidate PEP score. Fully calculating the validity of the WHO case definition would require data on false negatives (those turned away at screening who had EVD), but these data were not available. We compared the PEP score with the WHO case definition as accurately as the available data permitted for completeness (Technical Appendix).

To explore the potential effects of PEP scores on the number of correct and incorrect admissions at different times in the epidemic, we applied 2 PEP scores with different levels of sensitivity and specificity to 2 hypothetical populations of children: early in the epidemic when the proportion of suspected cases testing positive in Western EHUs was 77% (high background prevalence, October 2014); and later in the epidemic when the proportion was 4% (low background prevalence, March 2015). We used these hypothetical background prevalences with the sensitivity and specificity for each score to calculate number of true positives and negatives and false positives and negatives obtained by applying each score (Technical Appendix Tables 2–5) (*5*). We used multiple imputation by chained equations to account for missing data in the analysis of training and validation datasets (Technical Appendix) (*13*).

## Results

Of 1,054 children admitted with suspected cases to 11 EHUs during August 14, 2014–March 31, 2015, no result was available for 48 (5%) (Technical Appendix). Of the remaining 1,006 children, 309 (31%) were EVD positive and 697 (69%) EVD negative. Admissions rose from a median 8 (interquartile range [IQR] 5–11) per week in August–October 2014 to 50 (40–58) per week in February–March 2015, but the proportion of children that were EVD positive decreased from 57% (95% CI 43%–72%) in October 2014 to 6% (95% CI 2%–9%) in February 2015. At Ola During Children’s Hospital (ODCH), the main children’s hospital in Freetown, the onsite EHU received 59% of all EHU admissions, increasing from 12% in August–October 2014 to 82% in February–March 2015. 

We documented admission of 211 (21%) unaccompanied children. Data were missing for 297 (30%) of the children. EVD-positive children were more likely to be unaccompanied than those who were EVD negative (p<0.001).

Median patient age was 4 years (IQR 1.3–8.0 years), and 51% of the children were female (Table 1). Contact with EVD was reported for 275 (36%) of 754 children who had data available (75% of 1,006 total). Median time from symptom onset to hospital visit was 2 days (IQR 1–4). Fever data were available for 787 (78%) of children (Table 1), 775 of whom also had data available on the presence of >3 other symptoms. For those with data, fatigue/weakness was most frequently reported (97%), followed by fever (94%), anorexia (80%), vomiting (61%), headache (62%), and diarrhea (46%) (Table 1). Bleeding was rare, reported by 3%. Of the 809/1,006 (80%) of children who had sufficient symptom and contact history data recorded to ascertain if they fulfilled the late-2014 WHO suspected case definition, 31 (4%) were admitted despite not meeting the definition (Technical Appendix).

**Table 1:** Overview of 1,006 children who attended an Ebola holding unit and had EVD test results recorded, by final EVD test result status, Sierra Leone, August 14, 2014–March 31, 2015*

Characteristic	All children, no. (%) or median (IQR)	EVD negative			EVD positive		p value
No./no. available or median (IQR)	% (95% CI)	No./no. available or median (IQR)	% (95% CI)
Total†	1,006 (100)	697	69		309	31	–
Sex							
F	512 (51)	348/697	50 (46–54)		164/309	47 (41–53)	0.357
M	494 (49)	349/697	50 (46-54)		145/309	53 (47–59)	0.380
Median age, y (IQR)	4 (1.3–8)	3 (1–7)	–		6 (3–10)	–	<0.001
Age 0–2 y	392 (39)	336/697	48 (44–52)		56/309	18 (14–23)	<0.001
Positive contact, n = 754‡	275 (36)	108/541	20 (17–24)		167/213	78 (72–84)	<0.001
Days from symptoms to EHU admission, n = 772	2 (1–4)	2 (1–3)	–		3 (2–4)	–	0.001
Admitted with caregiver, n = 822	822 (82)	516/621	83 (80–86)		127/201	63 (56–70)	<0.001
Signs/symptoms§							
Fever, n = 787	740 (94)	528/566	93 (91–95)		212/221	96 (92–98)	0.160
Fatigue/weakness, n = 587	568 (97)	393/407	97 (94–98)		175/180	97 (94–99)	0.676
Vomiting/nausea, n = 777	472 (61)	345/556	62 (58–66)		127/221	57 (51–64)	0.238
Diarrhea, n = 763	351 (46)	252/548	46 (42–50)		99/215	46 (39–53)	0.988
Conjunctivitis, n = 669	152 (23)	73/463	16 (13–19)		79/206	38 (32–45)	<0.001
Anorexia, n = 779	621 (80)	452/560	81 (77–84)		169/219	77 (71–83)	0.269
Abdominal pain, n = 594	269 (45)	155/392	40 (35–45)		114/202	56 (49–63)	<0.001
Muscle pain, n = 577	212 (21)	127/377	34 (29–39)		85/200	43 (36–50)	0.037
Joint pain, n = 569	192 (34)	102/368	28 (23–33)		90/201	45 (38–52)	<0.001
Headache, n = 598	370 (62)	256/397	65 (60–69)		114/201	57 (50–64)	0.065
Difficulty breathing, n = 738	199 (27)	169/533	32 (28–36)		30/205	15 (10–20)	<0.001
Difficulty swallowing, n = 687	177 (26)	130/481	27 (23–31)		47/206	23 (17–29)	0.247
Rash, n = 728	98 (13)	88/522	17 (14–20)		10/206	5 (2–9)	<0.001
Cough, n = 587	70 (12)	57/407	14 (11–18)		13/180	7 (4–12)	0.019
Hiccups, n = 723	62 (9)	52/519	10 (8–13)		10/204	5 (2–9)	0.027
Unexplained bleeding, n = 726	22 (3)	19/518	4 (2–6)		3/208	1 (0–4)	0.114
Treatment¶							
Antimicrobial drug, n = 657	556 (85)	407/494	82 (79–86)		149/163	91 (86–95)	0.006
Antimalarial drug, n = 657	567 (86)	416/494	84 (81–87)		151/163	93 (87–96)	0.007
IV treatment	115 (11)	101/697	14 (12–17)		14/309	5 (2–7)	<0.001
Malaria RDT+, n = 74	33 (45)	31/57	54 (41–68)		2/17	12 (15–36)	0.002
Median days of EHU stay#	2 (1–3)	2 (1–2)	–		2 (1–3)	–	<0.001

*n values and denominators indicate no. children with recorded data available for variable (i.e., for binary variables children with neither “yes” nor “no” populated in their source notes were not included in the denominator, and for the median days symptoms to EHU admission variable those without date of start of symptoms were not included). EHU, Ebola holding unit; EVD, Ebola virus disease; RDT, rapid diagnostic test. †z-test of proportions, comparing whether the proportion of children with the variable was the same for EVD-negative and EVD-positive children (apart from numerical variables, for which a Wilcoxon rank-sum test was performed to test the hypothesis that the distribution of the variable was the same for EVD-negative and EVD-positive children).  ‡Total no. children admitted to holding units with test results available.  §Recorded on presentation at EHU.  ¶At EHU.  #Time from EHU admission until death, discharge, or transfer.

**Figure 2:**
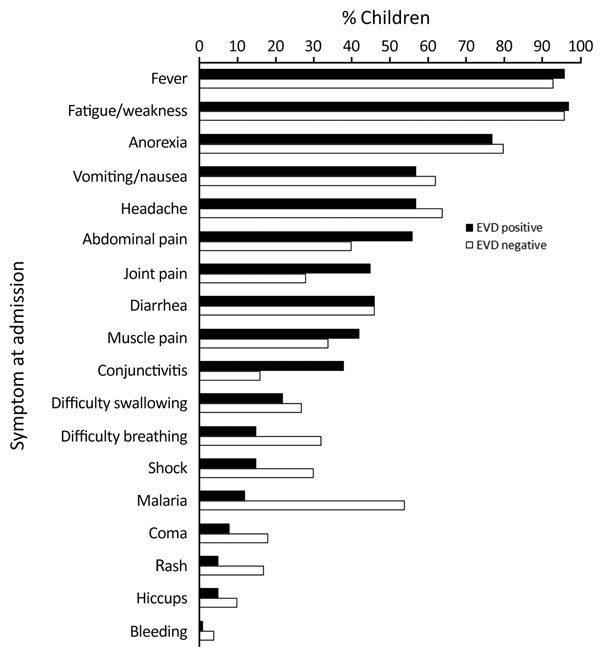
Frequency of clinical features in children positive and negative for Ebola virus disease (unadjusted) at an Ebola holding unit, Sierra Leone, August 14, 2014–March 31, 2015.

Children who were EVD negative were younger (median age 3 years [IQR 1–7 years] vs. 6 years [IQR 3–10 years]; p<0.001) (Table 1) and less likely to have conjunctivitis (p<0.001) than those who were EVD positive. Rash was more common in EVD-negative children (p<0.001) (Table 1; Figure 2). Similar proportions of both groups received antimicrobial and antimalarial drugs, and whereas both spent a median of 2 days in an EHU (admission to death or transfer/discharge), those with EVD tended to stay longer (p<0.001) (Table 1).

**Figure 3:**
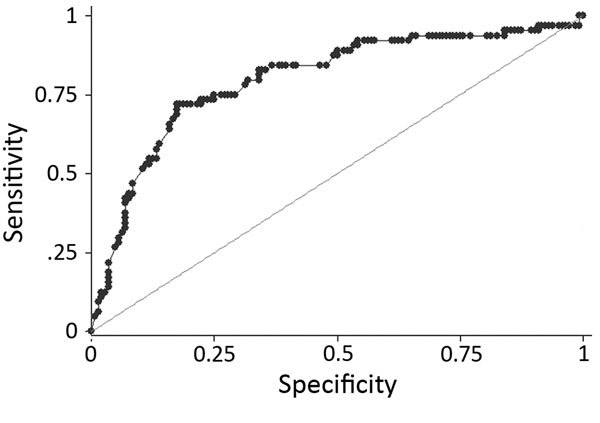
Receiver operating characteristics curve for final pediatric Ebola predictive score model based on a cohort of children who attended an Ebola holding unit and had Ebola virus disease test results recorded, Sierra Leone, August 14, 2014–March 31, 2015.

Randomly splitting the cohort of 1,006 children generated training and validation datasets of 504 and 502 (descriptive, crude, and adjusted analysis in Technical Appendix Table 6). In the training cohort, positive contact (multivariable OR 9.1, 95% CI 4.9–17); age >2 years (multivariable OR 2.9, 95% CI 1.4–5.8); and conjunctivitis (multivariable OR 3.8, 95% CI 1.9–7.8) were the strongest positive predictors of EVD. Headache, difficulty breathing, difficulty swallowing, and rash were negative predictors. The final multivariable predictive model included 12 variables: gender; age; positive contact; and presence or absence at hospital visit of fever, diarrhea, conjunctivitis, anorexia, abdominal pain, headache, difficulty breathing, difficulty swallowing, and rash. We present only analysis of the complete records, based on the similarity of receiver operating characteristics (ROC) curves for imputed and complete records analyses (Technical Appendix Table 7; Figure 3).

Assigning predictive model values derived from the training dataset to the validation dataset gave a range of PEP scores of 0–10. Plotting the ROC curve as sensitivity (x) against 1 − specificity (y) for all individual child PEP scores (with sensitivity and specificity calculated using the laboratory test as standard) demonstrated that the model had excellent discriminative ability (area under ROC curve = 0.80; Figure 3) (*14*). The model coefficients, p values, and assigned integer PEP scores are shown in Table 2 and the sensitivity, specificity, positive and negative predictive values, and percentage correctly classified for all possible PEP scores within the validation dataset in Table 3. A PEP score of 1 was 97% sensitive (95% CI 89%–100%) and 4% specific (95% CI 1%–8%), whereas the maximum PEP of 10 was 5% sensitive (95% CI 1%–13%) and 99% specific (95% CI 96%–100%) (Table 3).

**Table 2:** Scores for each of the variables included in Ebola pediatric predictive model

Variable	Coefficient (95% CI) from multivariable model	p value	Integer score value
Positive contact	2.21 (1.58–2.83)	<0.001	+2
Conjunctivitis	1.34 (0.62–2.05)	<0.001	+2
Age >2 y	1.06 (0.37–1.75)	0.003	+2
Fever	0.99 (–0.66 to 2.63)	0.241	+1
Anorexia	0.59 (–0.18 to 1.35)	0.133	+1
Male gender	0.49 (–0.11 to 1.08)	0.111	+1
Abdominal pain	0.42 (–0.23 to 1.08)	0.205	+1
Diarrhea	0.40 (–0.21 to 1.01)	0.197	+1
Difficulty breathing	–0.57 (–1.39 to 0.24)	0.168	−1
Difficulty swallowing	–0.59 (–1.39 to 0.19)	0.138	−1
Headache	–0.63 (–1.29 to 0.35)	0.063	−1
Rash	**−**1.00 (–2.13 to 0.14)	0.085	−2

**Table 3:** Validation of PEP score against a standard of laboratory-confirmed Ebola virus disease status, compared with WHO case definition, based on a cohort of children who attended an Ebola holding unit and had EVD test results recorded, Sierra Leone, August 14, 2014–March 31, 2015*

Score	Sensitivity (95% CI)	Specificity (95% CI)	PPV (95% CI)	NPV (95% CI)	% Correctly classified (95% CI)
0	100	1 (0–4)	31 (25–38)	100	31 (25–38)
1	97 (89–100)	4 (1–8)	31 (25–38)	71 (29–96)	32 (26–39)
2	97 (89–100)	13 (8–20)	33 (27–40)	91 (70–99)	39 (32–46)
3	94 (85–98)	30 (22–37)	37 (30–45)	91 (79–98)	49 (42–56)
4	86 (75–93)	49 (40–57)	43 (34–52)	89 (80–95)	60 (53–67)
5	77 (64–86)	67 (58–74)	51 (40–61)	87 (79–92)	70 (63–76)
6	58 (45–70)	82 (75–88)	59 (46–71)	81 (74–87)	75 (68–80)
7	44 (31–57)	92 (86–96)	70 (54–83)	79 (72–85)	77 (71–82)
8	23 (14–35)	95 (90–98)	68 (45–86)	74 (67–80)	73 (67–79)
9	11 (5–21)	98 (94–100)	70 (35–93)	71 (64–77)	71 (64–77)
10	5 (1–13)	99 (96–100)	75 (19–99)	70 (63–76)	70 (63–76)
WHO case definition†	98 (95–99)	5 (3–7)	30 (27–34)	84 (66–95)	33 (29–36)

*EVD, Ebola virus disease; NPV, negative predictive value; PEP, pediatric Ebola predictor; PPV, positive predictive value; WHO, World Health Organization. †Late-2014 WHO case definition with pediatric differentiations.

We considered the effect of using different PEP scores at different times during the outbreak. PEP score 3 (sensitivity of 94% and specificity of 30%) at the high background prevalence time point would have correctly classified 79 patients, with 16 EVD-negative patients admitted unnecessarily and 5 EVD-positive patients being incorrectly not admitted (Table 4; Technical Appendix Tables 2,3). Using a PEP score of 7 (sensitivity 44% and specificity 92%) at the low background prevalence time point would have correctly classified 90/100 patients, with 8 unnecessary admissions and 2 true EVD-positive patients incorrectly not admitted (Table 4; Technical Appendix Tables 4,5). Because we only have the true EVD status of patients who were admitted despite screening negative by WHO case definition (not the much larger number who were WHO case definition negatives and not admitted), the sensitivity and specificity calculated may be unreliable (Technical Appendix). However, on the basis of the data available, the WHO case definition was estimated to be 98% sensitive and 5% specific (Table 3; Technical Appendix Tables 8,9).

**Table 4:** Comparison of 2 different PEP scores on a hypothetical population of 100 suspected EVD patients at different points in EVD outbreak with differing prevalence of EVD*

PEP score	October 2014, 77% of suspected EVD+ cases†					March 2015, 4% of suspected EVD+ cases†			
True EVD+, correctly admitted	True EVD–, correctly not admitted	False EVD+, unnecessarily admitted	False EVD–, incorrectly not admitted	True EVD+, correctly admitted	True EVD–, correctly not admitted	False EVD+, unnecessarily admitted	False EVD–, incorrectly not admitted
3: 94% sensitivity, 30% specificity	72	7	16	5		4	28	68	0
7: 44% sensitivity, 92% specificity	34	21	2	43		2	88	8	2

*Laboratory-confirmed EVD status figures from Connaught Hospital (Freetown, Sierra Leone) during the 2014–2015 outbreak. EVD, Ebola virus disease; PEP, pediatric Ebola predictive; +, positive; –, negative.  †True or false EVD+ or EVD– determined by case ascertainment by PEP score. Admission result represents modeled outcome for patients in terms of Ebola holding unit.

## Discussion

This large, multicenter study compared symptoms at hospital visit in children <13 years old who were determined to be positive or negative for EVD during the outbreak in West Africa. As with many childhood diseases, EVD symptoms are nonspecific. The WHO indicators, including fever, breathing difficulties, and gastrointestinal symptoms, are common features in many pediatric pathologies. In this outbreak, gastrointestinal symptoms dominated, whereas bleeding, characteristic of previous outbreaks, was rare (*3*,*15*–*19*). This difference meant clinical diagnosis of EVD in the West African outbreak was difficult, which motivated this study. The lack of specificity of both early- and late-2014 WHO case definitions is highlighted by the fact that 69% of the children admitted as suspected EVD cases in this cohort were uninfected; that number increased to 94% in low-prevalence weeks (*10*).

Although elegant clinical predictive models have been developed for mixed-age cohorts, the focus of our model is children (*3*,*17*,*18*,*20*–*22*). The features at presentation that had the strongest association with a positive laboratory test result in this study were positive contact, conjunctivitis (similar to mixed-age cohorts [*17*,*22*]), and age >2 years. Fever, anorexia, abdominal pain, and diarrhea were weaker predictors of EVD. Certain features in the late-2014 WHO case definition were either not predictive or negative predictors, including bleeding, vomiting/nausea, difficulty breathing or swallowing, muscle or joint pain, headache, or rash (Table 1) (*9*). These findings emphasize the challenge of diagnosing EVD against high background rates of malaria and respiratory and gastrointestinal infections in children. The early-2014 WHO case definition demonstrated similar lack of specificity (32%) in 1 retrospective mixed-age cohort (sensitivity 80%) (*3*), although slightly better figures were documented in 2 smaller mixed-age cohorts (*20*,*23*).

The PEP score model described here could provide the basis for modifying pediatric case definitions as an outbreak evolves, or for different pediatric populations (e.g., at triage in an EHU vs. potentially lower-risk routine outpatient consultations). Similar to the mixed-age, malaria-sensitive score proposed by Hartley et al. (*17*), a patient with a high score would be strongly suspected and a low score weakly suspected of having EVD. In times of high community prevalence, children with a PEP score >7 (>92% specificity, 44% sensitivity) could rapidly be transferred to an ETC while awaiting laboratory confirmation, whereas those with a PEP score of 3 (sensitivity 94%, specificity 30%) could await test results in the EHU. This change could hasten access to specialist care for children with EVD and reduce exposure risk for those who are negative.

Assessing the applicability of our PEP score to future Ebola virus epidemics is important. Ideally, the model should be tested against other datasets from West Africa and prospectively in future outbreaks, because different EVD strains are likely to result in different disease manifestations. Indeed, in another pediatric cohort from Kailahun and Bo, Sierra Leone, containing 91 children <5 years of age, fever was absent in 25% (compared with 4% in our study) whereas bleeding was seen in 15% (*15*). In a large international cohort of 1,371 children <16 years of age with EVD, fever prevalence was 90% and bleeding 10% (*24*). However, it is possible that future pediatric case numbers may be smaller than those seen in this outbreak, which limits opportunities for prospective validation. We suggest governmental and nongovernmental organizations use this non–outbreak period to discuss with local stakeholders the acceptability of the trade-offs inherent within the PEP score, such as public health versus individual risk. One option would be the rapid setup of a triage facility admitting children with a PEP score >3 to await test results and fast-tracking those scoring >7 to specialized Ebola treatment. However, this decision is highly context-specific, and there are dangers in being too prescriptive without taking into account factors such as local healthcare-seeking behavior.

A key limitation to our study is that PEP scores are derived from a population of children admitted to EHUs, all of whom should have fulfilled either the early- or late-2014 WHO suspected case definition. We do not have information on those not admitted (who were either truly EVD negative or missed EVD-positive cases). Therefore, we could only use data on the small number of children admitted who did not meet the WHO case definition to calculate its sensitivity and specificity, and these children may not have been representative of children who were negative by the WHO case definition but not admitted. Our calculations of WHO case definition validity are therefore only included for completeness and must be treated with caution. A further limitation is reducing EVD contact to a binary variable; more in-depth information (such as whether the child has had contact with a dead body, or whether the child is breastfeeding) could give greater discrimination. However, because 37% EVD-positive children were unaccompanied at hospital admission, an in-depth contact history was unlikely to be reliable.

Missing and unreliable data are another limitation, illustrating the challenge of epidemiologic studies that analyze data from emergency settings. This study was retrospective, using data collected as part of outbreak data gathering rather than as part of a formal prospective study. We accounted for missing data using multiple imputation; reassuringly, imputed analysis gave similar results to a complete records analysis. We are also limited to data from those who sought medical care; thus, the description of EVD/non-EVD cases may be incomplete. External and prospective validation will be key but may be limited by small numbers. Finally, Hartley et al. have demonstrated the crucial importance of malaria testing in diagnostic screening for EVD (*17*). We did not have sufficient numbers of children with malaria test results in this cohort to incorporate malaria test results into our predictive score.

We have demonstrated that using a PEP score may help to streamline and improve management for children with suspected EVD, but the score still does not approach the accuracy of laboratory testing. Even by using a sensitive PEP score of 3, at high background prevalence, it is possible that 6% (5/77) of children with EVD could be turned away from an EHU in error (Table 4), which would have serious public health implications. Several highly sensitive rapid diagnostic tests (RDT) for EVD underwent preliminary testing toward the end of the West Africa outbreak, although the numbers of children included in these studies were limited (*25*,*26*). Judicious use of EVD RDTs coupled with PCR tests to confirm results could have reduced the scale of the Sierra Leone outbreak (*27*). Further development of RDTs, and guidance on selecting the children on whom to use them, is essential for preparing for and responding to future outbreaks. Incorporating screening criteria from an evidence-based clinical prediction model, such as this PEP score model, should contribute to this process.

In conclusion, this study compares features at hospital arrival in EVD-negative and EVD-positive children during the West African epidemic. We describe a predictive PEP score model that would allow for the selection of appropriate case definitions (prioritizing sensitivity or specificity) depending on the clinical and epidemiologic setting. The selected PEP scores had higher positive and negative predictive values than the current WHO case definition. Applying the score in combination with RDTs could be a successful strategy in future outbreaks. External validation of the PEP score will be key to establishing its utility, but because data are scarce, we suggest local stakeholders use this postoutbreak period to reflect how the PEP score might best be used in their context.

**Technical Appendix.** Additional information about Ebola infection in children and the development of the Pediatric Ebola Predictive Score (PEP score).
