# Supplementary material for: Development of a Pediatric Ebola Predictive Score, Sierra Leone
Source: Emerg Infect Dis. 2018 Feb;24(2):311–9. doi: 10.3201/eid2402.171018 (PMC5782873; doi:10.3201/eid2402.171018)
Supplement: Supplementary file 1 — Technical Appendix. Additional information about Ebola infection in children and the development of the Pediatric Ebola Predictive Score (PEP score). [file 17-1018-Techapp-s1.pdf]

Article DOI: https://doi.org/10.3201/eid2402.171018

Development of a Pediatric Ebola Predictive Score, Sierra Leone

Technical Appendix

Methods

Data Collection

The lead investigator (FF) carried out site visits to collect data from case investigation forms, site admission books, and other clinical records (e.g., hospital records at Ola During Children's Hospital) and to conduct staff interviews. Data were cross referenced with the Western Area Emergency Response Center (WAERC) database, which held demographic information used for coordinating bed management and transfers; district-wide laboratory results; child protection registers; burial records; and the database of the emergency telephone service for ambulance notification. Single data entry was carried out by FF and AN for expediency.

Patient Matching Schema

As previously described (*1*), a complete match consisted of the criteria below:

- Matching Western Urban Area (WUR) number and matching name. The WUR number was allocated with each case investigation form but was used inconsistently.
- Matching name, age, and case investigation form date.
- Four or more of these: name, age, case investigation form/laboratory test date, address, holding unit, eventual status (positive/negative/transferred/discharged).

A partial match consisted of $\geq$3 of name, age, CI form date, address, holding unit, eventual status (positive/negative/transferred/discharged). Small discrepancies in name spelling (e.g., Mohammed and Mohamed) could still be included as a complete match, but larger discrepancies of several letters (e.g., Abu and Abubakar) were a partial match. Matching was

performed by 2 investigators (M.G. and J.C.G.). Any discrepancies between the 2 investigators' categorization were raised with the lead investigator (F.F.), with whom the final decision rested. Partial matches were reviewed by the lead investigator and either discarded or included, depending on any additional information available (e.g., from telephone follow-ups). All complete matches were included in the analysis.

Predictive Model Building: Selection of Predictive Characteristics

After random splitting into the 2 equal-sized datasets for training and validation, we calculated the crude ORs of association between each potential predictive variable and outcome (EVD status). We prepared an initial multivariable model that included all potential predictive variables. Note that we included all variables in our initial multivariable model because selection of only those variables with a specific p-value from univariable results has the potential to wrongly reject important variables (*2*). We obtained a final training dataset model by removing variables with $p>0.3$ from the fully adjusted model in a backward-stepwise fashion. We used a relatively large p-value threshold (0.3) based upon guidance related to strategies for predictive modeling in small datasets (*3*). We considered the log odds of association for each variable the predictive model value for that variable.

Estimation of Validity of the Late-2014 WHO Case Definition

We provide an overview of how we calculated sensitivity, specificity, positive predictive value, negative predictive value, and number correctly identified for the WHO case definition (Technical Appendix Table 8, 9). A limitation of this approach is that we have only the "non-cases" that were admitted but (on the basis of the data we have), did not meet the WHO case definition. We do not have any data on the true EVD status of those that did not meet the WHO case definition and were not admitted. The proportion of EVD among those WHO non-cases who were not admitted may be different from the proportion of true EVD cases among those WHO non-cases who were admitted, which would affect the sensitivity and specificity estimates calculated in Technical Appendix Table 9.

Predictive Model Building: Handling of Missing Data

Missing data were assumed to be missing at random (MAR) (*1*), based upon analysis from a previous paper that used a subset of the same data analyzed in this study (*1*). Multiple imputation (MI) by chained equations was used to account for missing data in the analysis of the

training and validation datasets, with all variables with missing data from Table 2 included in the model plus the complete variables gender, age, date of admission and outcome status. For each dataset, 25 imputed datasets were created and combined for analysis, and comparisons of complete records analysis with imputed analysis results were made as appropriate. For the regression analysis, this involved tabulating the complete records crude ORs with the crude ORs from the imputed datasets. For the ROC analysis, this involved plotting 2 separate ROC curves: 1 using data only from those children in the validation dataset with complete records for all of the predictive model variables, and a second that averaged the sensitivity and specificity for each predictive model from across the 25 imputed datasets.

Results

Children without a Test Result Available

No test result was available for 48 children (5%). Of these, 8 (15%) had no outcome available. Thirty-one of the remaining 41 died, giving a CFR of 76%.

Children Admitted without Meeting Late 2014 WHO Suspect Case Definition

There were 197/1006 (20%) children with outcomes recorded who had data missing, meaning we were unable to assess if they met the late-2014 WHO case definition; 167 had both fever and contact status missing, 12 were missing contact status, 10 were missing fever, and 8 had insufficient symptom data. Of the remaining 809 children, 778 (96%) were classified as cases according to the WHO definition. Of the 31 (4%) that were not classified as cases, none had a positive contact reported; 29/31 had sufficient other symptoms recorded to meet the case definition but were recorded as not having fever, while the remaining 2 children were recorded as having a fever but did not have enough symptoms to meet the case definition.

Predictive Model Building and Validation

Regression Analyses

In developing the multivariable predictive model using the training dataset of 504 children, the symptoms fatigue/weakness and unexplained bleeding were not included in the regression analysis due to co-linearity with fever (fatigue/weakness) and insufficient numbers of events in EVD-positive children (unexplained bleeding). Crude analysis of complete records provided similar estimates to the imputed analysis (Technical Appendix Table 7).

ROC Analyses

After assigning predictive model coefficient values for each variable to children in both the imputed validation dataset and the complete records dataset, performing a comparison of model performance with laboratory confirmed EVD status demonstrated that the imputed and complete records ROC analyses produced similar curves and AUROCs (Technical Appendix Figure 3). Development of the (integer) PEP risk score and all subsequent assessments of sensitivity, specificity, and positive and negative predictive value were therefore performed on the data of the 206 children with complete records.

PEP Scores

Calculations of PEP scores against the standard, which is a blood-test result for EVD, are in Technical Appendix Tables 2–5. We based calculations on a hypothetical population of 100 suspected cases. We used known values of sensitivity and specificity of PEP score to calculate A, B, C, and D, as shown in Technical Appendix Table 10.

WHO Case Definition Performance

These numbers are from the 809 children for whom it was possible to ascertain whether they met the WHO case definition or not (i.e., had fever status recorded), and tabulated against their EVD test status.

First of all, consider only the 778 that met the WHO case definition. From this group, we can calculate a PPV of 30% (Technical Appendix Table 8). Now, include the 31 who were admitted but did not meet the case definition. This gives a total cohort of 809 children, and does allow calculation of the other measures of validity (Technical Appendix Table 9).

Calculations:

- Sensitivity = A / (A + C)
- Specificity = D / (D + B)
- Positive predictive value (PPV) = A / (A + B)
- Negative predictive value (NPV) = D / (D + C)
- Correctly identified = (A + D)/(A + B + C + D)

References

1. Fitzgerald F, Naveed A, Wing K, Gbessay M, Ross JC, Checchi F, et al. Ebola virus disease in children, Sierra Leone, 2014-2015. Emerg Infect Dis. 2016;22:1769–77. PubMed http://dx.doi.org/10.3201/eid2210.160579

2. Sun GW, Shook TL, Kay GL. Inappropriate use of bivariable analysis to screen risk factors for use in multivariable analysis. J Clin Epidemiol. 1996;49:907–16. PubMed http://dx.doi.org/10.1016/0895-4356(96)00025-X

3. Steyerberg EW, Eijkemans MJ, Harrell FE Jr, Habbema JD. Prognostic modeling with logistic regression analysis: in search of a sensible strategy in small data sets. Med Decis Making. 2001;21:45–56. PubMed http://dx.doi.org/10.1177/0272989X0102100106

4. Sterne JA, White IR, Carlin JB, Spratt M, Royston P, Kenward MG, et al. Multiple imputation for missing data in epidemiological and clinical research: potential and pitfalls. BMJ. 2009;338:b2393. PubMed http://dx.doi.org/10.1136/bmj.b2393

Technical Appendix Table 1. Modification of WHO screening symptom checklist for children ≤12 years*

Children <5 years	Children ≥5 years
Fever or history of fever within 48 hours	Fever or history of fever within 48 hours
Vomiting	Vomiting or nausea
Appetite loss	Appetite loss
Diarrhea	Diarrhea
Difficulty breathing	Difficulty breathing or swallowing
Excessive crying	Headache
Unexplained bleeding (nose, gums, gastrointestinal, or other)	Unexplained bleeding (nose, gums, gastrointestinal, or other)
Red eyes and or rash	Red eyes and or rash
Prostration	Weakness or severe fatigue
	Generalized muscular or articular pain
If fever (or history of fever) and ≥1 symptom, isolate child	If fever (or history of fever) and ≥3 symptoms, isolate child

*Source: Clinical Management of Patients in the Ebola Treatment Centres and Other Care Centres in Sierra Leone: A Pocket Guide. Interim emergency guidelines. Sierra Leone adaptation. World Health Organisation. December 2014.

Technical Appendix Table 2.Calculations using a specific predictive score cutoff for pediatric Ebola predictive score (PEP score) of 3 compared with blood test result, October 2014, Sierra Leone*

	EVD blood test result		
Category	Case (EVD+)	Non-case (EVD–)	Total
Case (score equal to or above predictive cutoff)	A = 72	B = 16	A + B = 88
Non-case (score below PEP cutoff)	C = 5	D = 7	C + D = 12
Total	A + C = 77	B + D = 23	A + B + C + D = 100

*77% EVD+ prevalence within suspected cases; sensitivity = 94%; specificity = 30%. EVD, Ebola virus disease.

Technical Appendix Table 3. Calculations using a specific predictive score cutoff for pediatric Ebola predictive score (PEP score) of 3 compared with blood test result, March 2015, Sierra Leone*

	EVD blood test result		
Category	Case (EVD+)	Non-case (EVD–)	Total
Case (score equal to or above predictive cutoff)	A = 4	B = 68	A + B = 72
Non-case (score below PEP cutoff)	C = 0	D = 28	C + D = 28
Total	A + C = 4	B + D = 96	A + B + C + D = 100

*4% EVD+ prevalence within suspected cases; sensitivity = 94%; specificity = 30%. EVD, Ebola virus disease.

Technical Appendix Table 4. Calculations using a specific predictive score cutoff for pediatric Ebola predictive score (PEP score) of 7 compared with blood test result, October 2014, Sierra Leone*

	EVD blood test result		
Category	Case (EVD+)	Non-case (EVD–)	Total
Case (score equal to or above predictive cutoff)	A = 34	B = 2	A + B = 36
Non-case (score below PEP cutoff)	C = 43	D = 21	C + D = 64
Total	A + C = 77	B + D = 23	A + B + C + D = 100

*77% EVD+ prevalence within suspected cases; sensitivity = 44%; specificity = 92%. EVD, Ebola virus disease.

Technical Appendix Table 5. Calculations using a specific predictive score cutoff for pediatric Ebola predictive score (PEP score) of 7 compared with blood test result, March 2015, Sierra Leone*

	EVD blood test result		
Category	Case (EVD+)	Non-case (EVD–)	Total
Case (score equal to or above predictive cutoff)	A = 2	B = 8	A + B = 10
Non-case (score below PEP cutoff)	C = 2	D = 88	C + D = 90
Total	A + C = 4	B + D = 96	A + B + C + D = 100

*4% EVD+ prevalence within suspected cases; sensitivity = 44%; specificity = 92%. EVD, Ebola virus disease.

Technical Appendix Table 6. Univariable and multivariable analysis of the association between child characteristics and laboratory-confirmed EVD-status for children (n = 504) who attended an Ebola holding unit in the western area of Sierra Leone*

Category	n (%)	No. EVD– (%)	No. EVD+ (%)	Crude OR (95% CI)†	Multivariable adjusted OR‡
Total§	504 (100)	349 (69)	155 (31)	-	-
Female	229 (45)	157 (69)	72 (31)	1	1
Male	275 (55)	192 (70)	83 (30)	0.94 (0.64–1.38)	1.62 (0.89–2.95)
Age, y					
Mean (SD)	4.6 (3.8)	3.8 (3.5)	6.5 (3.9)	1.19 (1.14–1.26)	-
Median (IQR)	4.0 (1.0–8.0)	2.0 (0.9–6.0)	6 (3–10)	*OR for each +1yr*	-
Age 2+ years	301 (60)	173 (57)	128 (43)	4.82 (3.03–7.68)	2.89 (1.44–5.77)
Positive contact (n = 373¶)	134 (36)	55 (41)	79 (59)	11.32 (6.82–18.78)	9.07 (4.85–16.97)
Days symptoms to admission (n = 391)					
Mean (SD)	3.2 (3.2)	3.2 (3.5)	3.2 (2.3)	1.00 (0.93–1.07)	–
Median (IQR)	2 (1–4)	2 (1–3)	3 (2–4)	*OR for each +1day*	–
Fever# (n = 398)	380 (95)	268 (71)	112 (29)	3.34 (0.76–14.78)	2.68 (0.52–13.93)
Fatigue/weakness** (n = 310)	299 (96)	209 (70)	90 (30)	-	–
Vomiting/nausea (n = 389)	234 (60)	174 (74)	60 (26)	0.67 (0.43–1.04)	–
Diarrhea (n = 387)	175 (45)	123 (70)	52 (30)	1.05 (0.68–1.63)	1.49 (0.81–2.74)
Conjunctivitis (n = 165)	84 (25)	38 (45)	46 (55)	3.88 (2.21–6.84)	3.80 (1.86–7.78)
Anorexia (n = 394)	317 (80)	229 (72)	88 (28)	1.57 (0.97–2.54)	1.80 (0.84–3.87)
Abdominal pain (n = 293)	129 (44)	77 (60)	52 (40)	1.66 (1.02–2.70)	1.53 (0.79–2.94)
Muscle pain (n = 286)	114 (40)	71 (62)	43 (38)	1.26 (0.76–2.10)	–
Joint pain (n = 284)	102 (36)	57 (56)	45 (44)	1.82 (1.06–3.12)	–
Headache (n = 302)	177 (59)	124 (70)	53 (30)	0.65 (0.41–1.02)	0.53 (0.27–1.04)
Difficulty breathing (n = 375)	105 (28)	89 (85)	16 (15)	0.35 (0.19–0.64)	0.56 (0.25–1.27)
Difficulty swallowing (n = 336)	91 (27)	70 (77)	21 (23)	0.52 (0.29–0.92)	0.55 (0.25–1.21)
Skin rash (n = 371)	52 (14)	46 (88)	6 (12)	0.30 (0.13–0.73)	0.37 (0.12–1.15)
Cough (n = 310)	44 (14)	36 (82)	8 (18)	0.47 (0.21–1.06)	–
Hiccups (n = 366)	38 (10)	33 (87)	5 (13)	0.30 (0.11–0.80)	–

Category	n (%)	No. EVD– (%)	No. EVD+ (%)	Crude OR (95% CI)†	Multivariable adjusted OR‡
Unexplained bleeding†† (n = 363)	12 (3)	11 (92)	1 (8)	–	–

*Analysis performed on the training dataset. EVD, Ebola virus disease; OR, odds ratio.
†Odds Ratio (95% confidence interval). Multiple imputation (MI) used to account for missing data for all estimates with missing data. MI model included all variables in this table with missing data (unless specified) plus the complete variables gender, age, date of admission and the outcome status.
‡Multivariable regression model included all variables with results in this column (with fever retained in the model on an a priori basis and all other variables selected for inclusion from a fully adjusted model using a backward stepwise approach, removing variables with p > 0.2) with MI applied.
§Number of children admitted to holding units.
¶Number of children with recorded data for variable.
#All symptoms in this table recorded upon arrival at EHU.
**Co-linear with fever in this dataset so not included in any regression analysis.
††Insufficient numbers for regression analysis.

Technical Appendix Table 7. Comparison of crude odds ratios obtained for the imputed dataset used in this study compared with a complete records approach for the training cohort*

		Crude odds ratio (95% confidence interval)	
Category	Value	Complete records†	Multiple imputation‡
Sex	Female	1	1
	Male	0.94 (0.64–1.38)	0.94 (0.64–1.38)
Age	OR increase per +1yr	1.19 (1.14–1.26)	1.19 (1.14–1.26)
Age (binary), y	0–2	1	1
	≥2	4.82 (3.03–7.68)	4.82 (3.03–7.68)
Positive contact	No	1	1
(missing n = 133)	Yes	12.30 (7.18–21.07)	11.32 (6.82–18.78)
Days symptoms to HU admission (missing n = 113)	OR per +1 d	1.00 (0.94–1.07)	1.00 (0.93–1.07)
		OR for each +1 day	*OR for each +1 day*
Fever	No	1	1
(missing n = 106)	Yes	3.34 (0.76–14.78)	3.34 (0.76–14.78)
Vomiting/nausea	No	1	1
(missing n = 115)	Yes	0.68 (0.44–1.06)	0.67 (0.43–1.04)
Diarrhea	No	1	1
(missing n = 117)	Yes	1.10 (0.70–1.71)	1.05 (0.68–1.63)
Conjunctivitis	No	1	1
(missing n = 165)	Yes	4.02 (2.39–6.76)	3.88 (2.21–6.84)
Anorexia	No	1	1
(missing n = 110)	Yes	0.85 (0.49–1.46)	1.57 (0.97–2.54)
Abdominal pain	No	1	1
(missing n = 211)	Yes	1.54 (0.95–2.50)	1.66 (1.02–2.70)
Muscle pain	No	1	1
(missing n = 218)	Yes	1.19 (0.73–1.95)	1.26 (0.76–2.10)
Joint pain	No	1	1
(missing n = 220)	Yes	1.69 (1.02–2.78)	1.82 (1.06–3.12)
Headache	No	1	1
(missing n = 202)	Yes	0.64 (0.40–1.04)	0.65 (0.41–1.02)
Difficulty breathing	No	1	1
(missing n = 129)	Yes	0.35 (0.20–0.64)	0.35 (0.19–0.64)
Difficulty swallowing	No	1	1
(missing n = 168)	Yes	0.58 (0.33–1.00)	0.52 (0.29–0.92)
Skin rash	No	1	1
(missing n = 133)	Yes	0.29 (0.12–0.70)	0.30 (0.13–0.73)
Cough	No	1	1
(missing n = 194)	Yes	0.47 (0.21–1.06)	0.47 (0.21–1.06)
Hiccups	No	1	1
(missing n = 138)	Yes	0.35 (0.13–0.91)	0.30 (0.11–0.80)

*Analysis performed on the training dataset. EVD, Ebola virus disease; OR, odds ratio.
†Complete records – only children with complete records for the variable in question were included in the analysis.
‡Multiple imputation (MI) used to account for missing data, with variables included in the MI model.

Technical Appendix Table 8. WHO case definition performance for patient-cases meeting the WHO case definition*

Category	EVD blood test result		
	Case (EVD+)	Non-case (EVD–)	Total
Case (score equal to or above predictive cutoff)	A = 237	B = 541	A + B = 778
Non-case (score below PEP cutoff)	C = 0	D = 0	C + D = 0
Total	A + C = 237	B + D = 541	A + B + C + D = 778

*Records were complete for 809 children; 778 met the WHO case definition. EVD, Ebola virus disease.
Sensitivity = A / (A + C)
Specificity = D / (D + B)
Positive predictive value (PPV) = A / (A + B) = 237/778*100 = 30%
Negative predictive value (NPV) = D / (D + C)
Correctly identified = (A + D)/(A + B + C + D)

Technical Appendix Table 9. WHO case definition performance for all patient-cases with complete records*

Category	EVD blood test result		
	Case (EVD+)	Non-case (EVD–)	Total
Case (according to WHO case definition)	A = 237	B = 541	A + B = 778
Non-case (according to WHO case definition)	C = 5	D = 26	C + D = 31
Total	A + C = 242	B + D = 567	A + B + C + D = 809

*Records were complete for 809 children; 778 met the WHO case definition. EVD, Ebola virus disease.
Sensitivity = A / (A + C) = 237/242*100 = 98%
Specificity = D / (D + B) = 26/567*100 = 5%
Positive predictive value (PPV) = A / (A + B) = 237/778*100 = 30%
Negative predictive value (NPV) = D / (D + C) = 26/31*100 = 84%
Correctly identified = (A + D)/(A + B + C + D) = 263/809*100 = 33%

Technical Appendix Table 10. Method used for calculating factors A – D for known values of sensitivity and specificity

Category	EVD blood test result		
	Case (EVD+)	Non-case (EVD–)	Total
Case (score equal to or above predictive cutoff)	A	B	A + B
Non-case (score below PEP cutoff)	C	D	C + D
Total	A + C	B + D	A + B + C + D

Sensitivity = A / (A + C) (*known value*)
Specificity = D / (D + B) (*known value*)
Positive predictive value (PPV) = A / (A + B)
Negative predictive value (NPV) = D / (D + C)
Correctly identified = (A + D)/(A + B + C + D)
Total=A + B + C + D – (*known value*)

Flowchart of patients attending Health Care Facilities in Freetown, Sierra Leone

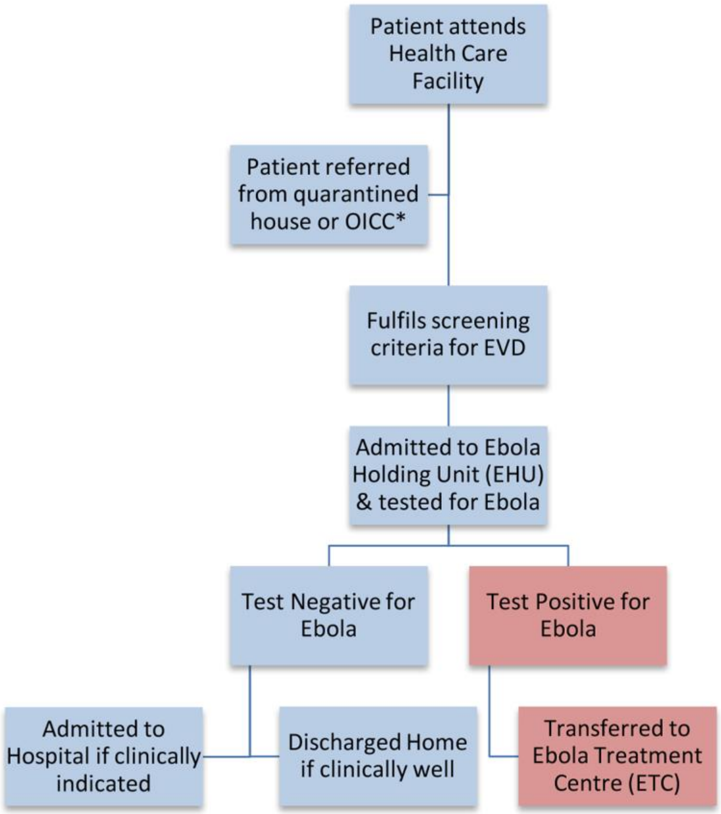

*OICC: Observational Interim Care Centre, set up to care for children with significant EVD exposure and no relatives to care for them for the duration of the 21 day incubation period. (Reproduced from Fitzgerald et al. under Creative Commons License[1])

Technical Appendix Figure 1. Flowchart of patients attending healthcare facilities in Freetown, Sierra Leone. Reproduced from Fitzgerald et al. under Creative Commons License.

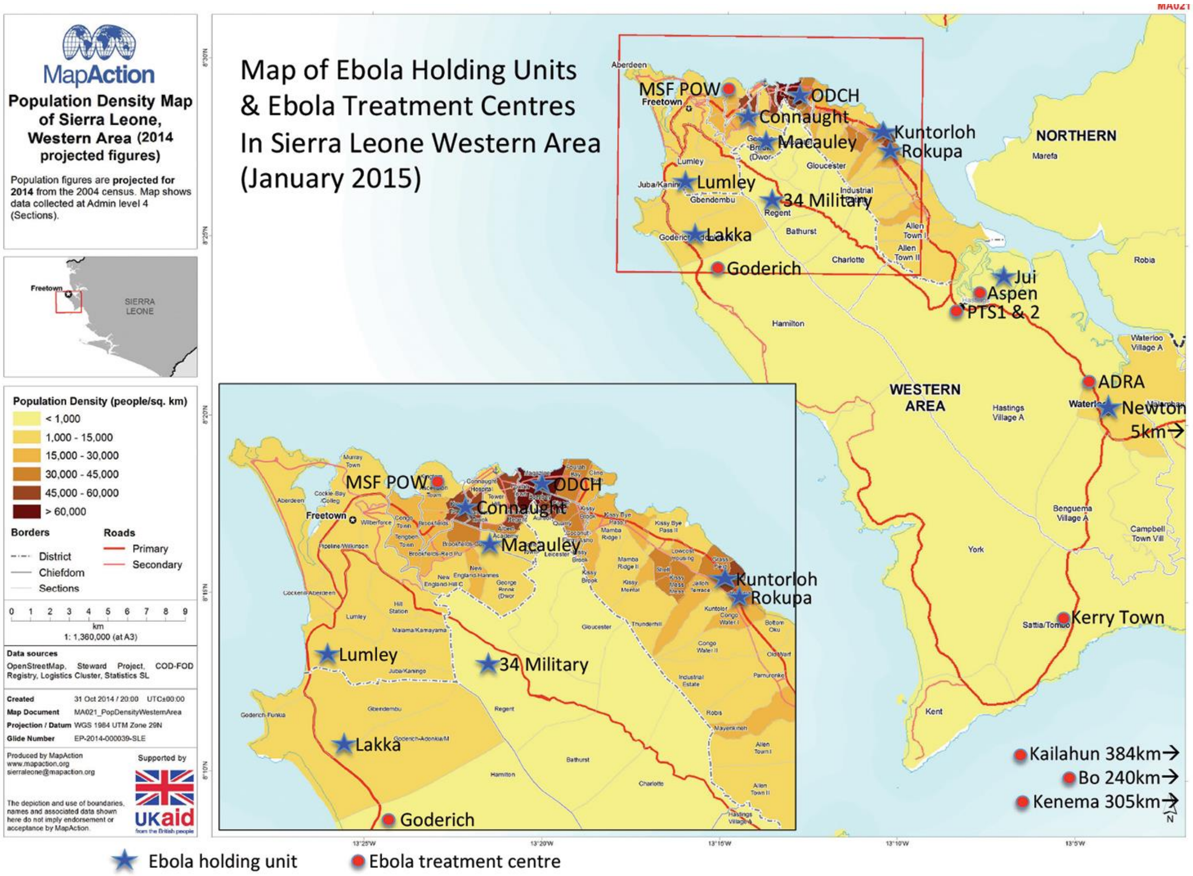

Technical Appendix Figure 2. Map of Western Area showing Ebola holding unit and Ebola treatment center locations as of January 2015, superimposed on population density map. Reproduced from Fitzgerald et al. under Creative Commons License.

Receiver Operating Characteristics (ROC) curve for different Paediatric Ebola Prognostic Scores – comparison of complete records (blue) with imputed data (red)

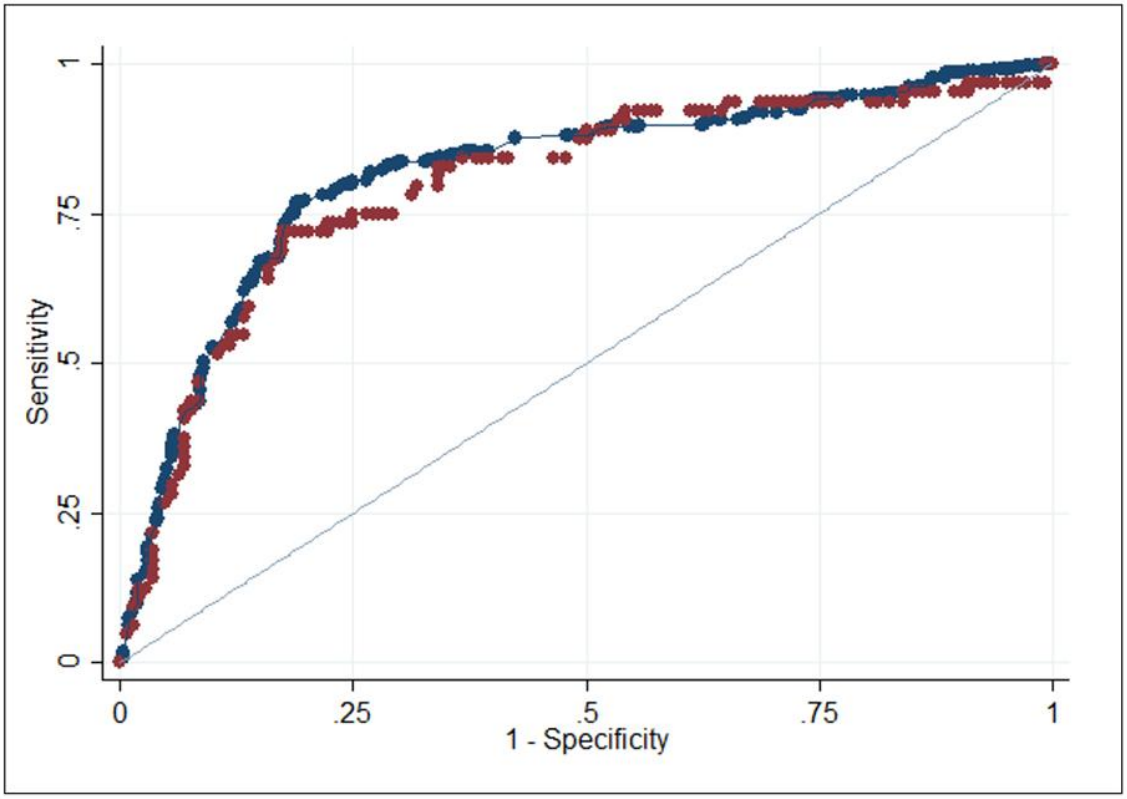

Technical Appendix Figure 3. Receiver Operating Characteristics (ROC) curve for different Pediatric Ebola Predictive Scores calculated from a comparison of complete records with imputed data.
